# The Long-Term Outcome in a Cohort of 52 Patients With Symptomatic Intramedullary Spinal Cavernous Hemangioma After Microsurgery and Emergency Rescue Surgery

**DOI:** 10.3389/fmed.2022.872824

**Published:** 2022-04-25

**Authors:** Yu Duan, Renling Mao, Xuanfeng Qin, Yujun Liao, Jian Li, Gong Chen

**Affiliations:** ^1^Department of Neurosurgery, Huadong Hospital, Fudan University, Shanghai, China; ^2^Department of Neurosurgery, Huashan Hospital, Fudan University, Shanghai, China

**Keywords:** intramedullary spinal cavernous hemangioma, spinal cord, spinal cavernous hemangioma, central nervous system, prognosis, microsurgery, salvage therapy

## Abstract

**Background:**

Surgery is the mainstay treatment for patients with symptomatic intramedullary spinal cavernous hemangioma (ISCH), however the time of surgical intervention remains controversial. In this study, we proposed emergency rescue surgery (ERS) for patients in deteriorative type. The prognostic factors of patients with ISCH after microsurgery and the clinical effect of ERS were analyzed.

**Methods:**

From January 2013 to November 2019, 52 patients with symptomatic ISCH treated by microsurgical treatment were collected, ranging in age from 17 to 66 years old (mean: 45.8 ± 13.5 years). The course of the disease ranged from 2 days to 20 years. Of 52 lesions, 17 lesions were in the cervical segment, 25 in the thoracic segment, and 10 in the lumbosacral segment; while seven cases were at the ventral surface, 25 cases at the dorsal surface, and 20 cases at the central spinal cord. The sagittal diameter ranged from 1 to 58 mm (median: 17.3 mm). The transverse diameter ratio ranged from 20 to 80% (median: 50.7%). Thirty-two patients were diagnosed as deteriorative type and 22 were treated by ERS.

**Results:**

At 12 months after surgery, all patients were followed up, and no residual or recurrence was found in all patients. Twenty-five patients (48.1%) showed spinal cord functional improvement after surgery; 25 (48.1%) had no functional change; 2 (3.8%) got worse. For deteriorative patients, ERS group had a significantly higher improvement rate than the non-ERS group (χ^2^ = 5.393, *P* = 0.02); For all 52 patients, the factors as a lesion at the ventral surface (Z = 10.453, *P* = 0.015), or lumbosacral segment (χ^2^ = 9.259, *P* = 0.010) and longer course of disease (Z = −2.021, *P* = 0.043) were potential risks in functional recovery in univariate analysis; and in multiple-factor analysis, the lesion at the lumbosacral segment (OR = 4.004, 95% CI: 1.341~11.961, *P* = 0.013) was the independent risk factors for the functional recovery.

**Conclusions:**

Microsurgical resection is safe and effective for symptomatic ISCH. The ERS is an effective way to improve deteriorative patients' spinal cord function at long-term follow-up. The lesion at the lumbosacral segment is one of the poor prognostic factors.

## Introduction

Intramedullary spinal cavernous hemangioma (ISCH) is an uncommon spinal vascular disease, accounting for 5–15% of spinal vascular malformations (1–3). The natural history of symptomatic ISCH is not completely understood, although most ISCH has a benign clinical course, the annual rate for a first hemorrhage could be up to 4.5% per year and the annual rate for recurrent hemorrhage would be up to 66% (4).

For asymptomatic or small (1–3 mm) ISCH, conservative treatment might be optimal due to surgery-related complications (5, 6), and the patients with hemorrhagic cavernomas should consider surgical intervention, which prevents recurrent hemorrhage and further neurologic deterioration (7–11). The duration means the time from the onset of symptoms to surgery, which varies greatly at different centers, from several hours to several decades (12, 13). Some surgeons believe it is best to allow the neurological symptoms to plateau, to prevent further damage to viable tissue (14); while others believe the risk of rebleeding is too high to wait (15, 16). With the advancement of surgical skills and the continuous accumulation of experience, surgical excision is more active, and duration has been constantly shortening, and the patients with the shorter duration of presurgical symptoms (≤ 3 months) have better clinical outcomes. However, the most surgeon still do not take operation timing seriously, and there are still few studies about the relationship between operation timing and clinical prognosis.

We believe that if surgical resection and laminectomy are performed as soon as possible, it will effectively alleviate spinal edema and avoid deterioration of spinal function. In 2013, according to our new clinical classification, we proposed emergency rescue surgery (ERS) for treating patients in deteriorative type. The present study was conducted to evaluate long-term outcomes in a cohort of 52 patients with symptomatic ISCH after microsurgery and to study the clinical effects of deteriorative patients with ERS.

## Materials and Methods

### Patients and Study Design

From January 2013 to January 2019, the patients with symptomatic ISCH in two neurosurgery centers were analyzed. Inclusion criteria: 1. Diagnosed by ISCH surgically and pathologically; and 2. Over 14 years old. Exclusion criteria: 1. Recurrence of ISCH after surgery; 2. Multiple ISCHs with brain function impaired; 3. Extramedullary (roots) lesions; 4. After radiotherapy (e.g., gamma knife); and 5. Loss to follow-up. After the exclusion of 16 cases, 52 patients (28 males and 24 females) were included. The onset age ranged from 15 to 80 years (mean: 45.8 ± 14 years) and the course of disease ranged from 2 days to 20 years (median: 12 days). Twenty-eight patients suffered from muscle weakness or dyskinesia, 34 patients suffered from paresthesia (20 cases felt pain, and 14 felt numb); 33 patients suffered from bowel and/or bladder dysfunction. Nine patients (17.3%) had multiple intracranial or intramedullary ISCHs, and 6 patients (11.5%) had a familial history of ISCH.

### Preoperative MRI or DSA

All patients were examined by MRI scan and enhancement. Spinal angiography would be performed to exclude other types of vascular malformations if necessary. There were 17 lesions in the cervical segment, 25 lesions in the thoracic segment, and 10 lesions in the lumbosacral segment. Sagittal length: 1~62 mm, with an average of 15.8 ± 9.8 mm. Horizontal transverse diameter ratio (maximum diameter at the horizontal position of the lesion/spinal cord diameter of the lesion): 18%~80% (49.4% ± 16.8); Horizontal position: seven cases were in ventral surface, 25 cases on the dorsal surface, and 20 cases in center.

### Clinical Course Classification

**Figure 1:**
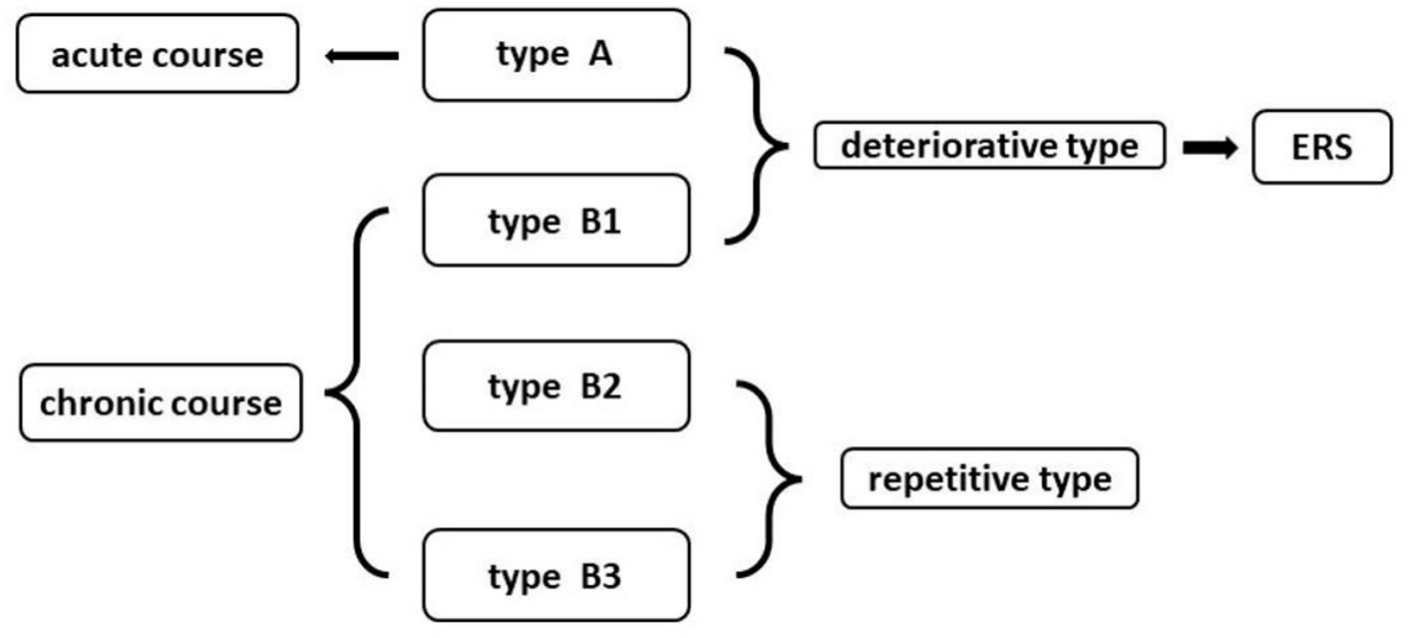
The new clinical classification of intramedullary spinal cavernous hemangioma (ISCH) and its relationship with emergency rescue surgery (ERS).

The new clinical classification was based on Ogilvy types (15). In this study, four subtypes (A1, B1, B2, B3, and B4) were divided into the acute course (Type A) and chronic course (Type B). Type A: acute onset of symptoms with rapid decline; Type B1: repeating deterioration of neurological decline with acute onset; Type B2: acute onset of mild symptoms with subsequent gradual decline lasting weeks to months; Type B3: discrete episodes of neurological deterioration with varying degrees of recovery between episodes. Types A and B1 are defined as acute and chronic deteriorative types, respectively. Types B2 and B3 are defined as chronic repetitive types (Figure 1).

### Emergency Rescue Surgery (ERS)

The patients with deteriorative types were suggested by ERS (Figure 1). The ERS was defined as: time interval between the day of the first acute onset to the day of operation is <3 days for patients with Type A, and the time interval between the last acute onset to the day of operation is <7 days for patients with Type B1.

### Surgical Key Point

**Figure 2:**
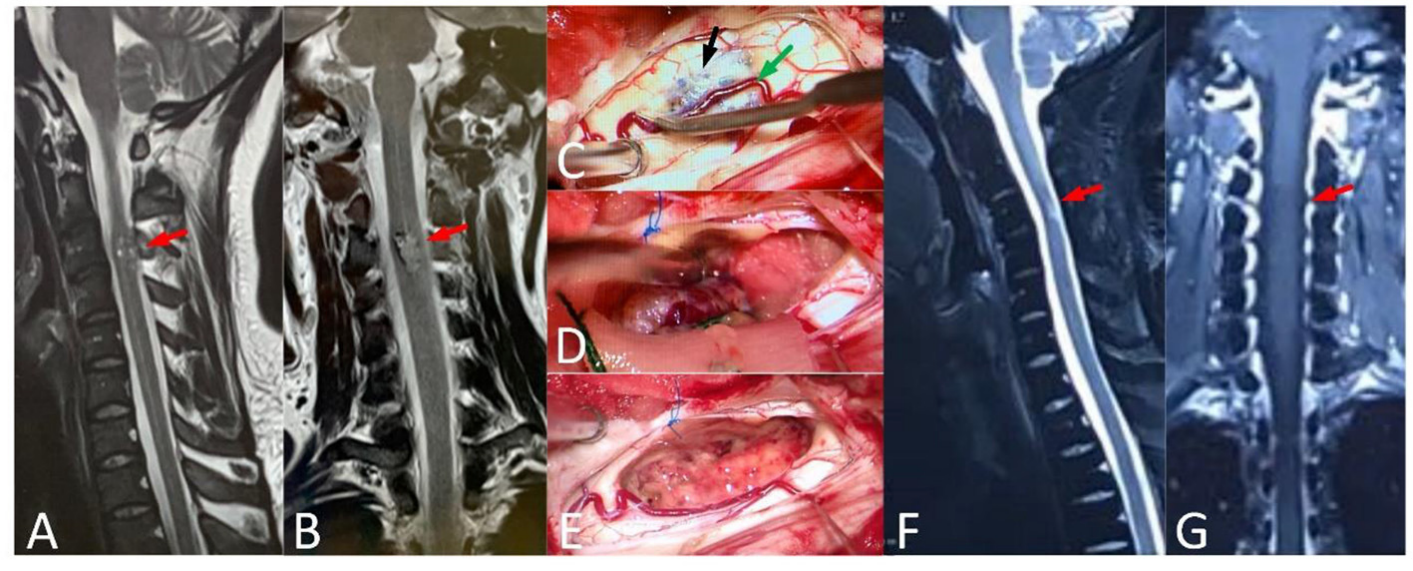
A 37-year-old male, with numbness of limbs and trunk for 5 years, and standing difficulty for 5 days (mALs = 13 points, severe disorder, Type A), was treated by ERS. **(A,B)** Preoperative spinal MRI examination revealed ISCH in C3 (red arrow). **(C–E)** The lesion was visible on spinal cord surface (black arrow) and the artery (green arrow) was protected carefully. **(F,G)** MRI re-examination showed no residual or recurrence in the operative area at 4 years after surgery and the mAS was 7 points at the last follow-up.

**Figure 3:**
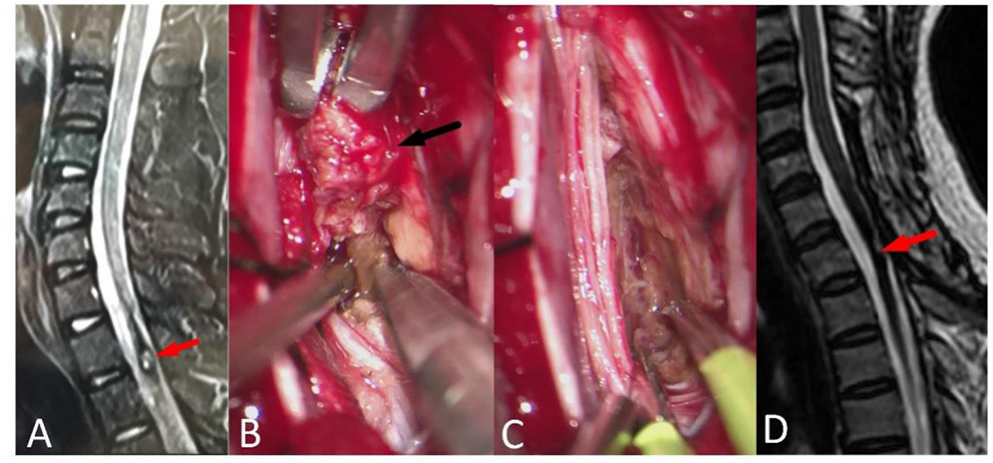
A 15-year-old female with walking difficulty for 2 months and aggravating for 5 days (mALs = 12 points, severe disorder, Type B1), was treated by ERS. **(A)** Preoperative spinal MRI examination revealed ISCH in T1-T2 (red arrow). **(B,C)** The lesion was resected in pieces (black arrow) by posterior central approach. **(D)** MRI reexamination showed no residual or recurrence at 2 years after surgery and the mAS was 8 points at the last follow-up.

According to location, different approaches were adopted. If the lesion was visible on the surface, it could be removed directly (Figure 2). If the lesion was close to the center and in deep, the posterior midline approach was adopted (Figure 3). Somatosensory-evoked potentials (SEPs) and motor-evoked potentials (MEPs) were monitored during surgery.

### Neurofunctional Assessment and Follow-Up

The patient's spinal cord function was evaluated by modified Aminoff-Logue scale (mAL)-excellent: normal or normal, ≤ 2 points; mild disorder, 3 ~ 5 points; moderate disorder, 6~8; severe disorder, ≥9 points. The improvement or degradation of neurological function was defined when the score was changed at least one grade and in the excellent group, the improvement was defined by patients' subjective feeling or limb muscle strength improving. Clinical follow-up was conducted after 12 months and every one year after surgery, including MRI examination and mALs assessment.

### Statistical Analysis

The SPSS23.0 statistical software package was used to process data. Fisher's exact test was used for the comparison of the rates between groups. The normal distribution measurement data (showed by mean and standard deviation) were tested by the *t*-test and the non-normal distribution measurement data (showed by median and quartile spacing) were tested by the rank-sum test (Mann-Whitney *U*-test). The improvement of factors at the neurological function was analyzed by multivariate logistic regression analysis. The *P* < 0.05 was considered statistically significant.

## Results

### Postoperative Imaging

Patients have received an MRI plain scan and enhanced at follow-up, and no residual or recurrence of ISCH was found in the operative area at all 52 patients at the last follow-up.

### ERS

Among the 32 patients with deteriorative type, the average time interval between the day of onset to the day of surgical intervention was 12.2 ± 17.9 days (2~90 days), 22 patients were treated by ERS, and 10 patients did not receive ERS. In ERS group, 16 (68.8%, 22/32) patients showed neurofunctional improvement at long-term follow-up, and in the non-ERS group, only 2 (20%, 2/10) patients showed improvement (Table 1). There was a significant difference in improvement rate between the two groups (χ^2^ = 7.767, *P* = 0.005).

**Table 1:** Clinical data between ERS group and non-ERS group.

	**ERS group (*n =* 22)**	**Non-ERS group (*n =* 10)**	**Test value**	***P* value**
Age (mean ± SD, years)	46.4 ± 14.8	47.2 ± 9.1	2.362^a^	0.878
Male (*n*,%)	10 (46.7%)	8 (80.0%)	3.334^b^	0.068
Deteriorative type				
Acute deteriorative type (*n*,%)	16	6	0.518^b^	0.472
Chronic deteriorative type (*n*,%)	6	4		
Segment			1.715^b^	0.424
Cervical segment	7	2		
Thoracic segment	10	7		
Lumbosacral segment	5	1		
Horizontal position			4.368^b^	0.113
Dorsal surface	12	8		
Center	6	3		
Ventral surface	0	3		
Transverse diameter ratio (%)	49% (40%-55.3%)	50% (45.3%-64%)	−0.636^c^	0.535
Sagittal length (mm)	12 (11-20)	15.0 (12.8-17.0)	−0.986^c^	0.324
Family history (*n*,%)	1 (6.7%)	1 (8.3%)	0.027^b^	0.869
Multiple lesions (*n*,%)	1 (6.7%)	1 (8.3%)	0.027^b^	0.869
Preoperative mALs	8 (3-12)	3 (2.8-10.3)	−1.555^c^	0.120
Onset to operation (d)	3 (3-6)	15 (11-37)	−4.606^c^	<0.001
Prognosis			7.767^b^	0.005
Improvement	16	2		
No change	5	7		
Deterioration	1	1		

a *T value,*

b *χ^2^ value;*

c *Z value. The normal distribution measurement data were showed by mean and standard deviation and the non-normal distribution measurement data were showed by median and quartile spacing*.

### The Prognostic Factors After Microsurgical Intervention

Of 52 patients, eight patients (15.4%, 8/52) showed a decrease in mAL score after surgery, but most were transient and six had recovered to the preoperative state within two months after surgery. At 12 months after surgery, 48.1% (25/52) patients showed improvement, 48.1% (25/52) had no changed and 3.8% (2/52) got worse at mAL score. Lesion in ventral surface (Z = 10.453, *P* = 0.015), at lumbosacral segment (χ^2^ = 9.259, *P* = 0.010) and longer course of disease (Z = −2.021, *P* = 0.043) were potential poor factors in functional recovery in univariate analysis. In multiple-factor analysis, the lesion at lumbosacral segment (OR = 4.004, 95% CI: 1.341~11.961, *P* = 0.013) were the independent risk factors for the functional recovery (Table 2).

**Table 2:** Univariate analysis and multi-factor regression analysis of spinal cord function recovery after operation in 52 patients with ISCH.

**Variable**	**Univariate analysis**				**Multi-factor logistic regression anlysis**	
	**Outcome**		**Test value**	***P value***	**Odds ratio (95% confidence interval)**	***P value***
	**Improve (23)**	**No-Improve (25)**				
Age (mean ± SD, years)	41.9 ± 15.7	49.4 ± 12.7	−1.999^a^	0.051		
Male (*n*, %)	11 (44.0%)	17 (63.0%)	1.878^b^	0.171		
Clinical presentation			2.720^b^	0.437		
Type A	13	9				
Type B1	5	5				
Type B2	4	9				
Type B3	3	4				
Segment			9.259^b^	0.010	4.004 (1.341~11.961)	0.013
Cervical segment	12	5				
Thoracic segment	12	13				
Lumbosacral segment	1	9				
Horizontal position			6.264^b^	0.044	1.457 (0.805~2.636)	0.213
Dorsal surface	16	9				
Center	8	12				
Ventral surface	1	6				
Transverse diameter ratio (%)	50% (40–57%)	56% (30–66%)	−0.018^c^	0.985		
Sagittal length (mm)	15 (11–19.5)	14 (11.8–30)	−0.350^c^	0.727		
Family history (*n*,%)	3 (12.0%)	1 (7.1%)	0.23^b^	0.632		
Multiple lesions (*n*,%)	3 (12.0%)	2 (14.3%)	0.042^b^	0.838		
Onset to operation (d)	7 (3–25)	10 (5–80)	−2.021^c^	0.043	1.001 (0.998~1.004)	0.409
Preoperative mALS	4 (3–12)	3 (2–12)	−0.080^c^	0.936		

a *T value,*

b *χ^2^ value;*

c *Z value*.

*The normal distribution measurement data were showed by mean and standard deviation and the non-normal distribution measurement data were showed by median and quartile spacing*.

### Two Cases With Postoperative Aggravation

Case 1-A 63 years old male suffering from the sudden loss of muscle strength and bowel and urine dysfunction for 12 days. The lesion was located at T11~T12 by MRI test. The patient was judged to Type A by our new clinical classification and evaluated at 12 points by mAL scale before surgery. During the surgery, the amplitude is permanently <20% by electrophysiologic monitoring. After 1 month of surgery, spinal cord function decreased to 13 points and had not improved at the last follow-up.

Case 2-A 50 years old male suffering from episodic and recurrent pain in both lower limbs for 1 year. The lesion was located at T12 by MRI test and was evaluated at eight points by mAL scale before surgery and judged to Type B3 by our new clinical classification. During the surgery, the amplitude of electrophysiologic monitoring had no abnormity. After 1 month of surgery, spinal cord function decreased to 11 points and had improved at 10 points 1 year after surgery.

## Discussion

Surgery is the mainstay treatment for ISCH (18), which can eliminate the risk of subsequent hemorrhage (19), and prevent further neurological decline (20). However, the timing for surgery has been argued for decades (20). In this study, we firstly proposed ERS intervention for ISCH patients with clinical progression and further confirmed most patients could benefit from ERS compared with non-ERS. As we believe, a wide range of symptoms to either an acute hemorrhage forming a space-occupying lesion, or by edema can lead to a progressive or acute decline in neurological function (3, 21, 22), and choose to evacuate the clot early to relieve compression (17, 23); whereas, another surgeon still believed the timing should be postponed for several weeks because it would help resolve the hematoma, diminishing spinal cord swelling, and creating a discrete border on the lesion itself (12).

According to previous literature, the median duration of primary symptoms to referral was 6.5 months (24), the mean duration from primary symptoms to subsequent hemorrhage or deteriorative symptoms was 1.42 years and the mean duration from primary symptoms to surgery was 2.1 years (25). It means those patients with deteriorative symptoms may not be treated by microsurgery timely at most neurosurgical centers. For meta-analysis research, earlier timing for surgery was beneficial for neurological function (26) and Zhang reported that most pediatric patients presented with acute symptoms and they can benefit from surgery at the acute phase of neurological deterioration (27). In this study, the longer course of the disease was also one of the potential negative factors for recovery. From 2013, the ERS system for patients with symptomatic ISCH has been built at our two neurosurgical centers, cooperating with departments of emergency, imaging, and surgery, specifically for deteriorative patients (A and B1). In this study, 16 patients (68.8%) in ERS group showed neurofunctional improvement, and the rate at the non-ERS group was only 20%, which verified ERS could be beneficial for recovery of deteriorative type patients. There were also 10 patients with deterioration who not receive ERS, seven patients were delayed at the referral process, and the other three patients were indecisive about ERS and treated by microsurgery at a routine time. As we think, the treatment system of ERS needs constantly perfecting, such as letting more primary hospitals and patients with symptoms understand the clinical characteristics and treatment of ISCH.

**Figure 4:**
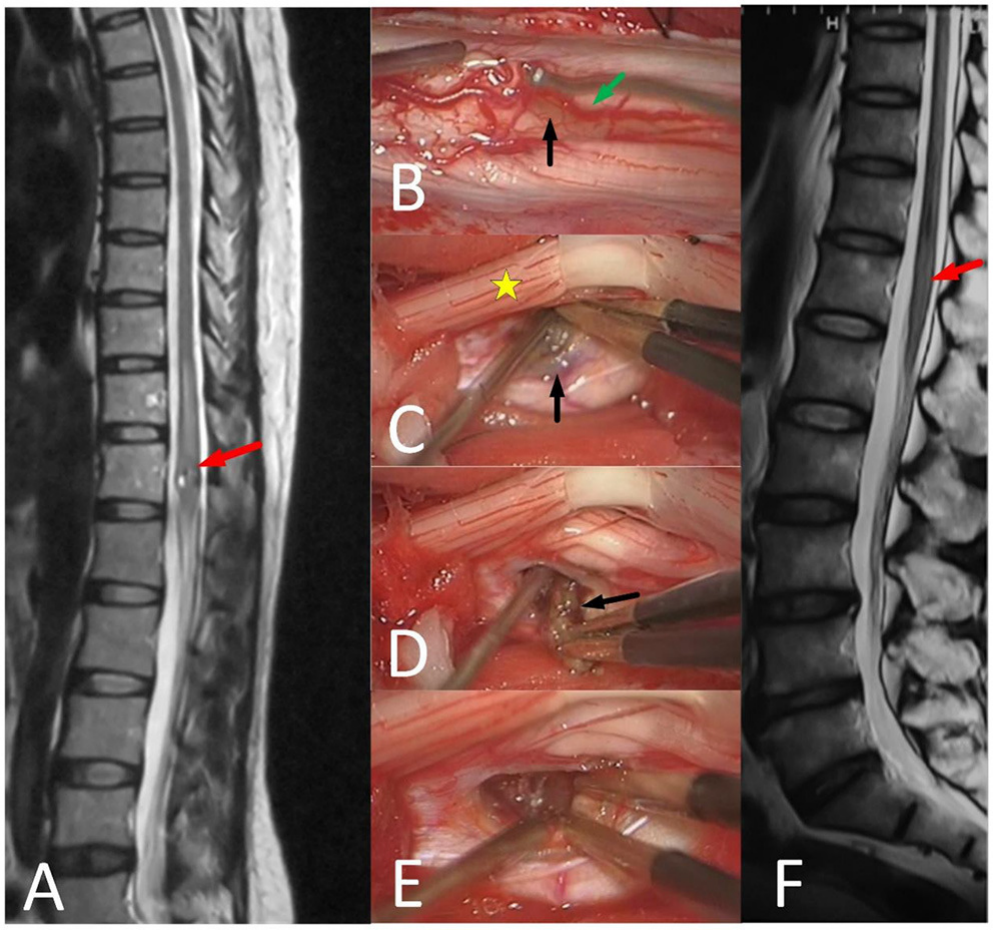
51-year-old female with unsteady walking for 6 months and urinating difficulty for 3 days (mALs = 11 points, severe disorder, type B1), was treated by ERS. **(A)** Preoperative spinal MRI examination revealed ISCH in ventral conus (red arrow). **(B–E)** A The brown lesion on the spinal cord surface (black arrow) could be seen after pushing posterior nerve roots (yellow star) aside and the artery (green arrow) was protected carefully. **(F)** MRI reexamination showed no residual or recurrence at 3 years after surgery and the mAS was 10 points at the last follow-up.

Many worsening predictors after resection have been reported, such as poor preoperative function, thoracolumbar-level lesions, and the depth of lesions (28, 29). In our study, lesions in the ventral surface, at a lumbosacral segment, and a longer course of disease were potential predictors for poor functional recovery. For intramedullary ventrolateral deep lesion, Ren adopted a new surgical approach, the dorsal root entry zone myelotomy (DREZ), and showed that of 10 patients, two (20%) patients improved and eight (80%) patients were stable after the new approach (30). Ginalis reported a multi-segment, hemorrhagic intramedullary cavernous malformation from C7 to T3 was resected through a lateral myelotomy approach at the site of superficial hemorrhage (31). As we believe, the reason for DREZ or a lateral myelotomy approach being chosen, is because the corridor is the closest way into the lesion. Westphal reported 500 cases of intramedullary lesions, including ependymomas, astrocytoma, vascular pathologies, indicating that safe and complete removal can be achieved by posterior midline approach (32). The posterior midline approach for deeper lesions and the direct approach for superficial lesions are our two conventional approaches: 1. It needs to be emphasized that 1 blood vessels on the surface of the spinal cord should be carefully protected during the operation (Figure 4); 2. Avoid pulling and twisting the spinal cord; and 3. Try not to use bipolar coagulation, and if necessary, keep its energy to the minimum.

In this study, electrophysiologic monitoring was performed in all patients, including motor-evoked potentials (MEPs) and somatosensory-evoked potentials (SEPs). As our plan, if the amplitude is <50%, the operation should be suspended (33). Compared with Li's result, of the 52 patients with ISCH under electrophysiologic monitoring, 17 patients showed permanent changes, two had long-term residual neurologic deficits (34). In our cohort, ten patients showed transient amplitude decline, and the other two patients showed permanent changes according to electrophysiologic monitoring. During 1 week after surgery, eight patients (15.4%, 8/52) showed a decrease in function, and functional impairments included hypoesthesia in six patients, sphincter dysfunction in two patients, and decreased muscle strength in two patients. Most of the functional impairments were transient, six had recovered to the preoperative state within two months after surgery, and two with lesions at the lumbosacral segment. At 12 months after surgery, only two patients got worse at sphincter dysfunction or decreased muscle strength than the status before surgery, whose lesions were all located at the lumbosacral segment, which meant that recovery is more difficult in those patients with lesions lumbosacral segment.

## Conclusions

Microsurgical resection is safe and effective for symptomatic ISCH; however, lumbosacral lesions had a poor prognosis. The patients with a deteriorative type would receive a better prognosis at long-term follow-up if treated by ERS.

## Data Availability Statement

The raw data supporting the conclusions of this article will be made available by the authors, without undue reservation.

## Ethics Statement

The studies involving human participants were reviewed and approved by Huashan Hospital and Huadong Hospital, affiliated to Fudan University. Written informed consent was obtained from the individual(s) or their legal guardian/next of kin to participate in this study and for the publication of any potentially identifiable images or data included in this article.

## Author Contributions

GC proposed ERS theory and performed the operations. YD wrote the article and analyzed the data. RM, XQ, and YL assisted to finish part of the operations and collected the data. JL was responsible for intraoperative neurophysiological monitoring. All authors contributed to the article and approved the submitted version.

## Funding

This work was supported by Project of Shanghai Science and Technology Committee (18411962400).

## Conflict of Interest

The authors declare that the research was conducted in the absence of any commercial or financial relationships that could be construed as a potential conflict of interest.

## Publisher's Note

All claims expressed in this article are solely those of the authors and do not necessarily represent those of their affiliated organizations, or those of the publisher, the editors and the reviewers. Any product that may be evaluated in this article, or claim that may be made by its manufacturer, is not guaranteed or endorsed by the publisher.
